# Pharmacokinetics, safety and efficacy of elvitegravir/cobicistat/emtricitabine/tenofovir alafenamide in children with HIV aged from 2 years and weighing at least 14 kg

**DOI:** 10.1002/jia2.26414

**Published:** 2025-01-30

**Authors:** Eva Natukunda, Aditya H. Gaur, Pope Kosalaraksa, Elizabeth Hellström, Renate Strehlau, Afaaf Liberty, Stephanie Cox, Rory Leisegang, Ramesh Palaparthy, Susanne Crowe, Vinicius Vieira, Kathryn Kersey, Natella Rakhmanina

**Affiliations:** ^1^ Department of Paediatrics Joint Clinical Research Centre Kampala Uganda; ^2^ Department of Infectious Diseases St. Jude Children's Research Hospital Memphis Tennessee USA; ^3^ Department of Pediatrics Faculty of Medicine Khon Kaen University Khon Kaen Thailand; ^4^ Be Part Yoluntu Centre Paarl South Africa; ^5^ Department of Paediatrics and Child Health University of the Witwatersrand Johannesburg South Africa; ^6^ Chris Hani Baragwanath Hospital Johannesburg South Africa; ^7^ Gilead Sciences, Inc. Foster City California USA; ^8^ Children's National Hospital Washington DC USA; ^9^ The George Washington University Washington DC USA; ^10^ Elizabeth Glaser Pediatric AIDS Foundation Washington DC USA

**Keywords:** antiretroviral therapy, children, E/C/F/TAF, elvitegravir, HIV‐1, tenofovir alafenamide

## Abstract

**Introduction:**

Elvitegravir/cobicistat/emtricitabine/tenofovir alafenamide (E/C/F/TAF) was efficacious and well tolerated in children/adolescents with HIV (aged ≥6 years, weighing ≥25 kg) in a Phase 2/3 study. Here, we report data from children aged ≥2 years and weighing ≥14–<25 kg.

**Methods:**

This is an analysis of data from the youngest cohort in an open‐label, multicentre, multi‐cohort, single‐group, international study of children/adolescents with HIV. Participants in this cohort were children aged ≥2 years, weighing ≥14–<25 kg at screening and able to swallow tablets, on stable antiretroviral therapy with virologic suppression (HIV‐1 RNA <50 copies/ml for ≥6 consecutive months) and a CD4 count ≥400 cells/µl. Eligible participants received low‐dose E/C/F/TAF (90/90/120/6 mg) once daily through Week 48. The study included pharmacokinetic evaluation of the low‐dose E/C/F/TAF tablet at Week 2. Safety, efficacy, palatability and acceptability were also evaluated.

**Results:**

Between 16 January and 25 November 2019, 27 participants were enrolled with a median (quartile [Q]1, Q3) age of 6 (4, 8) years, body weight of 19.3 (17.0, 20.5) kg, CD4 count of 1061 (895, 1315) cells/µl and CD4 cell percentage of 37.4 (30.6, 40.3). Most (92.6%) participants acquired HIV through vertical transmission. On 6 October 2020 (data‐cut), median (Q1, Q3) exposure to E/C/F/TAF was 48.3 (48.0, 60.1) weeks. Pharmacokinetic parameters were within the safe and efficacious range of previous data in adult and paediatric populations. Drug‐related treatment‐emergent adverse events occurred in 4/27 (15%) participants. There were no Grade 3/4 adverse events, or adverse events leading to E/C/F/TAF discontinuation. One participant experienced a serious treatment‐emergent adverse event (Grade 2 pneumonia not considered E/C/F/TAF related). Virologic suppression (US FDA Snapshot algorithm) was maintained by 26/27 (96%) participants at Weeks 24 and 48. At Week 48, most children reported positive palatability (84.6%) and acceptability (96.2%).

**Conclusions:**

These data support the use of single‐tablet E/C/F/TAF (90/90/120/6 mg) regimen for the treatment of HIV in children aged ≥2 years and weighing ≥14–<25 kg.

**Clinical Trial Number:**

NCT01854775

## 1 INTRODUCTION

In 2023, there were an estimated 1.4 million children <15 years of age with HIV worldwide, of whom only 57% received antiretroviral therapy (ART) [1, 2]. Without ART, 50% of children with HIV will die before they reach 2 years of age, and 80% will die before they are 5 [3].

For children with HIV, ART options remain limited, and treatment adherence can be particularly challenging. The ability to use tablets depends on a child's age/weight and developmental stage [4].

Elvitegravir/cobicistat/emtricitabine/tenofovir alafenamide (E/C/F/TAF) is a once‐daily single‐tablet regimen comprising an integrase strand transfer inhibitor (INSTI), elvitegravir (EVG), boosted with a CYP3A4 inhibitor, cobicistat (COBI), and two nucleoside reverse transcriptase inhibitors: emtricitabine (FTC) and tenofovir alafenamide (TAF). E/C/F/TAF is approved for use in children and adolescents weighing ≥25 kg and adults in the United States and Europe, at a dose of 150/150/200/10 mg, and for children aged ≥2 years and weighing ≥14–<25 kg in Europe at a dose of 90/90/120/6 mg [5, 6]. This low‐dose tablet was formulated to provide exposures equivalent to the adult dose in younger children.

In a Phase 2/3 study in ART‐naïve adolescents aged ≥12–<18 years (Cohort 1) and virologically suppressed children aged ≥6–<12 years, weighing ≥25 kg (Cohort 2), E/C/F/TAF (150/150/200/10 mg) demonstrated high rates of virologic suppression and was well tolerated over 48 weeks [7, 8]. Low‐dose E/C/F/TAF (90/90/120/6 mg) was also investigated in virologically suppressed children aged ≥2 years and weighing ≥14–<25 kg at screening (Cohort 3). Here, we report the pharmacokinetics (PK), safety and efficacy of the low‐dose tablet in Cohort 3 over 48 weeks.

## 2 METHODS

### 2.1 Study design and participants

GS‐US‐292‐0106 (NCT01854775) is a Phase 2/3, open‐label, multicentre, multi‐cohort, single‐group study ongoing at sites in South Africa, Thailand, Uganda, the United States (all cohorts) and Zimbabwe (Cohort 3) [7, 8].

Participants in Cohort 3 were children with HIV aged ≥2 years, weighing ≥14–<25 kg at screening and able to swallow tablets, on stable ART with virologic suppression (HIV‐1 RNA <50 copies/ml for ≥6 consecutive months) and a CD4 count ≥400 cells/µl.

Participants had an estimated glomerular filtration rate (eGFR) ≥90 ml/min/1.73 m^2^ and no suspected HIV resistance to any components of E/C/F/TAF.

The study was conducted in accordance with the principles of the Declaration of Helsinki. Parents or guardians provided written informed consent for all participants; where applicable, participants also provided age‐appropriate assent.

### 2.2 Study treatments

Eligible participants received oral E/C/F/TAF at the lower dose (90/90/120/6 mg) as a 16 × 7 mm‐sized tablet. Tablets were administered once daily through Week 48. Participants who attained a weight of ≥25 kg during the study were switched to the adult‐dose tablet.

### 2.3 Procedures

PK, safety and efficacy were assessed. Adverse events (AEs) were coded using the Medical Dictionary for Regulatory Activities (version 23.0); AEs and laboratory abnormalities were graded according to the Gilead Sciences Grading Scale for Severity of Adverse Events and Laboratory Abnormalities, adapted from the Division of AIDS guidelines (version 1.0, December 2004; updated, August 2009). HIV‐1 RNA plasma concentration was measured using the COBAS^®^ AmpliPrep/COBAS^®^ TaqMan^®^ HIV‐1 Test, version 2.0 (Roche Diagnostics, Pleasanton, California, USA).

Intensive PK evaluation was performed at Week 2, with samples collected ≤0.5 hours before dosing and at 0.25, 0.5, 1, 1.5, 2, 3, 4, 5 and 8 hours post‐dose. For all analytes, plasma concentrations were measured using high‐performance liquid chromatography–tandem mass spectrometry (QPS, Newark, Delaware, USA).

Lumbar spine (LS) and total‐body‐less‐head (TBLH) bone mineral density (BMD) was evaluated at baseline, Week 24 and Week 48 using dual‐energy X‐ray absorptiometry.

### 2.4 Outcomes

The co‐primary endpoints were area under the concentration–time curve over the dosing interval (AUC_tau_) for EVG and TAF, and incidence of treatment‐emergent AEs (TEAEs) through Week 24. Secondary endpoints included observed plasma drug concentration at the end of the dosing interval (C_tau_) for EVG; maximum observed plasma drug concentration (C_max_) for EVG and TAF; and AUC_tau_, C_max_ and C_tau_ for COBI, FTC and tenofovir (TFV). Efficacy endpoints included the percentage of participants with HIV‐1 RNA <50 copies/ml (US FDA Snapshot algorithm), and change from baseline in CD4 count and percentage at Weeks 24 and 48. Other endpoints included the incidence of TEAEs and treatment‐emergent serious AEs through Week 48.

### 2.5 Statistical analyses

A sample size of 25 participants was estimated to provide 90% power to demonstrate exposure equivalence for EVG AUC_tau_ and TAF AUC_tau_ between children aged ≥2 years, weighing ≥14–<25 kg, and historical data in adults, assuming the following: (1) the expected geometric least squares mean (GLSM) ratios for EVG AUC_tau_ and TAF AUC_tau_ in children and adults would be 1; (2) the bioequivalence boundaries were 70% and 143%; (3) two one‐sided statistical tests would be performed, each at an alpha level of 0.05; and (4) inter‐participant standard deviations (natural log scale) for EVG AUC_tau_ and TAF AUC_tau_ were 0.34 and 0.52 h·ng/ml, respectively. Intensive PK assessments were performed in all participants who received ≥1 dose of study drug and who had any non‐missing key PK parameter for the respective analyte from the intensive PK evaluation at Week 2. All PK parameters were estimated in non‐compartmental analyses (WinNonlin^®^ software, version 6.3).

Safety and efficacy were analysed in all participants who received ≥1 dose of study drug.

BMD standard Z‐scores were computed based on a chronological age‐matched population of the same sex and ethnicity. Height‐for‐age Z‐scores (HAZs) and body mass index (BMI) Z‐scores were derived from the Centers for Disease Control and Prevention growth charts for the United States [9]. BMD Z‐scores were then adjusted for HAZs. If a participant's age (or HAZ) was outside the BMD reference data for Z‐scores, the Z‐score was determined by extrapolation.

Demographics, baseline disease characteristics and other outcomes were summarized using descriptive statistics.

## 3 RESULTS

### 3.1 Baseline demographics and disease characteristics

Cohort 3 enrolled 27 children between 16 January and 25 November 2019. Participants were predominantly female (63%) and Black (89%), with a median age of 6 (quartile [Q]1, Q3: 4, 8; min, max 3, 9) years, and a median body weight of 19.3 (Q1, Q3: 17.0, 20.5; min, max: 14.6, 23.5) kg. All participants were virologically suppressed at baseline and 92.6% had acquired HIV through vertical transmission; median (Q1, Q3) CD4 cell count was 1061 (895, 1315) cells/µl and CD4 cell percentage was 37.4 (30.6, 40.3). Prior to switching to E/C/F/TAF, 100% received nucleoside reverse transcriptase inhibitors, 74.1% protease inhibitors, 22.2% non‐nucleoside reverse transcriptase inhibitors and 3.7% INSTIs. On 6 October 2020 (data cut‐off), median (Q1, Q3) exposure to E/C/F/TAF was 48.3 (48.0, 60.1) weeks.

### 3.2 PK of the low‐dose E/C/F/TAF tablet

PK parameters of the low‐dose E/C/F/TAF (90/90/120/6 mg) tablet are summarized in Table 1. AUC_tau_ GLSMs for TAF and EVG were 93% and 39% higher than in adults with HIV‐1 receiving adult‐dose E/C/F/TAF; corresponding C_max_ values were 50% and 43% higher. AUC_last_ GLSM for TAF was 51% higher. As TAF undergoes rapid intracellular clearance, TAF C_tau_ could not be determined. C_tau_ GLSM for EVG was numerically lower than in adults. All exposure parameters for TFV were similar to those observed in adults. For COBI, the AUC_tau_ GLSM was 37% higher than in adults, while C_max_ and C_tau_ were comparable between children and adults. For FTC, the C_tau_ GLSM was similar to that observed in adults, while the AUC_tau_ and C_max_ GLSMs were 61% and 39% higher than in adults, respectively.

**Table 1:** Pharmacokinetic parameters of the low‐dose E/C/F/TAF (90/90/120/6 mg) tablet in children and adult‐dose E/C/F/TAF (150/150/200/10 mg) tablet in adults (historical data) derived from standard non‐compartmental analyses

	Children aged ≥2 years weighing ≥14–<25 kg^a^		Adults (reference)^b^		
	*n*	GLSM	*n*	GLSM	% GLSM ratio of children: reference (90% CI)
Elvitegravir (E)					
AUC_tau_ (h·ng/ml)	24	29,864.03	19	21,553.74	138.56 (111.93, 171.52)
C_max_ (ng/ml)	27	2850.88	19	1997.55	142.72 (112.94, 180.35)
C_tau _(ng/ml)	22	195.43	19	247.71	78.90 (53.13, 117.17)
Cobicistat (COBI)					
AUC_tau_ (h·ng/ml)	21	12,262.48	19	8975.72	136.62 (103.01, 181.20)
C_max_ (ng/ml)	27	1274.56	19	1400.19	91.03 (71.04, 116.64)
C_tau_ (ng/ml)	18	16.61	18	17.01	97.64 (64.57, 147.66)
Emtricitabine (F)					
AUC_tau_ (h·ng/ml)	27	18,620.48	19	11,576.55	160.85 (143.01, 180.90)
C_max_ (ng/ml)	27	2808.24	19	2014.35	139.41 (120.28, 161.59)
C_tau_ (ng/ml)	27	77.40	19	89.11	86.86 (72.30, 104.34)
Tenofovir alafenamide (TAF)^c^					
AUC_tau_ (h·ng/ml)	17	343.54	539	178.30	192.68 (165.97, 223.67)
AUC_last_ (h·ng/ml)	27	268.88	539	178.30	150.80 (119.16, 190.85)
C_max_ (ng/ml)	27	217.75	539	144.88	150.29 (115.68, 195.27)
Tenofovir (TFV)					
AUC_tau_ (h·ng/ml)	27	326.70	841	283.86	115.09 (106.96, 123.84)
C_max_ (ng/ml)	27	19.07	841	14.79	128.95 (119.46, 139.20)
C_tau_ (ng/ml)	27	11.14	841	10.30	108.12 (100.11, 116.78)

Abbreviations: AUC_last_, area under the plasma concentration–time curve from time zero to the last quantifiable concentration; AUC_tau_, area under the plasma concentration–time curve over the dosing interval; CI, confidence interval; C_max_, maximum observed plasma drug concentration; C_tau_, observed plasma drug concentration at the end of the dosing interval; E/C/F/TAF, elvitegravir, cobicistat, emtricitabine and tenofovir alafenamide; GLSM, geometric least squares mean.

^a^ Pharmacokinetic parameters for Cohort 3 are derived from the intensive pharmacokinetic analysis set.

^b^ Adult pharmacokinetic parameters for elvitegravir, cobicistat and emtricitabine are derived from the intensive pharmacokinetic analysis set in a Phase 2 study (GS‐US‐292‐0102); data for tenofovir alafenamide and tenofovir were from population pharmacokinetic analyses in Phase 3 studies (GS‐US‐292‐0104 and GS‐US‐292‐0111).

^c^ C_tau_ was not calculated for tenofovir alafenamide due to its short half‐life [6].

### 3.3 Safety, efficacy, palatability and acceptability of the low‐dose E/C/F/TAF tablet

In total, 20/27 (74.1%) participants experienced a TEAE up to the data cut‐off date. Most TEAEs were Grade 1 (mild) in severity and not considered related to study drug (Table 2). The most common TEAE was upper respiratory tract infection. Three (11.1%) participants experienced drug‐related vomiting and/or diarrhoea, and one participant (3.7%) experienced asthenia. There were no renal TEAEs (including renal tubulopathy/Fanconi syndrome), Grade 3 or 4 TEAEs, or TEAEs leading to study drug discontinuation. One participant (3.7%) experienced a treatment‐emergent serious AE of Grade 2 pneumonia, which was not considered related to study drug and resolved within 4 days. No treatment‐emergent deaths were reported before the data cut‐off date. One participant (3.7%) experienced a treatment‐emergent Grade 3 laboratory abnormality of decreased platelets. No Grade 4 abnormalities occurred.

**Table 2:** Cumulative TEAEs through Week 48 in children receiving the low‐dose E/C/F/TAF tablet (safety analysis set)

	Children aged ≥2 years weighing ≥14–<25 kg (*n* = 27)
Any TEAE, *n* (%)	20 (74.1)
Any related to study drug	4 (14.8)
Related to study drug and occurring in >1 participant^a^	
Vomiting	2 (7.4)
Diarrhoea	2 (7.4)
Any Grade 3 or 4 TEAE, *n* (%)	0
Related to study drug	0
Any serious TEAE, *n* (%)	1 (3.7)
Related to study drug	0
Discontinuations due to TEAEs, *n* (%)	0
Deaths, *n* (%)	0

Abbreviations: E/C/F/TAF, elvitegravir, cobicistat, emtricitabine and tenofovir alafenamide; TEAE, treatment‐emergent adverse event.

^a^ One participant experienced both vomiting and diarrhoea.

Overall, LS and TBLH BMD increased relative to baseline during follow‐up, while LS and TBLH HAZ‐adjusted BMD Z‐scores remained relatively stable (Table 3). Changes from baseline in median eGFR and BMI Z‐score were small.

**Table 3:** Key bone, renal and metabolic parameters in children receiving the low‐dose E/C/F/TAF tablet

	LS		TBLH				
	BMD, g/cm^2^, median (Q1, Q3) (*n* = 27)	HAZ‐adjusted BMD Z‐score, median (Q1, Q3) (*n* = 27)	BMD, g/cm^2^, median (Q1, Q3) (*n* = 27)	HAZ‐adjusted BMD Z‐score, median (Q1, Q3) (*n* = 27)	eGFR, ml/min/1.73 m^2^, median (Q1, Q3) (*n* = 27^b^)	Body weight, kg, median (Q1, Q3) (*n* = 27)	BMI Z‐score, median (Q1, Q3) (*n* = 27)
Baseline	0.46 (0.40, 0.48)	−1.60 (−2.02, −0.85)	0.51 (0.45, 0.57)	−1.39 (−1.81, −0.78)	147.0 (139.1, 159.1)	19.3 (17.0, 20.5)	−1.24 (−1.79, −0.02)
Change at Week 24^a^	4.23 (−0.21, 6.52)	0.21 (−0.16, 0.36)	3.42 (2.13, 6.45)	0.08 (−0.24, 0.35)	4.1 (−13.8, 26.8)	1.1 (0.5, 1.8)	0.16 (−0.20, 0.48)
Change at Week 48^a^	4.58 (1.86, 9.86)	0.14 (−0.14, 0.36)	6.51 (4.89, 10.26)	−0.06 (−0.33, 0.27)	9.6 (−11.2, 19.3)	2.6 (2.0, 3.2)	0.41 (−0.19, 0.85)

Abbreviations: BMD, bone mineral density; BMI, body mass index; E/C/F/TAF, elvitegravir, cobicistat, emtricitabine and tenofovir alafenamide; eGFR, estimated glomerular filtration rate; HAZ, height‐for‐age Z‐score; LS, lumbar spine; Q, quartile; TBLH, total‐body‐less‐head.

^a^ Presented as percentage change for BMD measurements and absolute change for HAZ‐adjusted BMD Z‐scores, eGFR measurements (calculated according to the Schwartz formula), body weight and BMI Z‐scores.

^b^ Week 24: *n* = 19; Week 48: *n* = 27.

High rates of virologic suppression were maintained from baseline. At both Weeks 24 and 48, 26/27 (96.3%) participants had HIV‐1 RNA <50 copies/ml, one participant (3.7%) was viraemic (HIV‐1 RNA <1000 copies/ml) and no participants had missing data. Based on pill counts, their adherence was >90% at the viraemic visits; however, transitory low adherence cannot be ruled out. No resistance to study drug was detected, and the participant subsequently resuppressed on study drug. The median absolute CD4 count decreased from 1061 cells/µl at baseline to 941 and 883 cells/µl at Weeks 24 and 48, respectively. CD4 percentage remained stable (37.4% at baseline, 34.0% and 36.3% at Weeks 24 and 48, respectively). At Day 1 and Week 48, respectively, most children swallowed the tablets whole (81.5%, 96.3%), and reported positive palatability (77.8%, 84.6%) and acceptability (90.9%, 96.2%).

## 4 DISCUSSION

The results of this ongoing open‐label study in virologically suppressed children aged ≥2 years and weighing ≥14–<25 kg show that PK parameters of the low‐dose E/C/F/TAF tablet were within the safe and efficacious range. Moreover, 48 weeks of treatment with the low‐dose E/C/F/TAF tablet was well tolerated and maintained virologic suppression.

PK of drugs in children vary by age, weight and developmental stage; therefore, establishing the correct dose to ensure efficacy without impacting safety is important [10]. Exposures derived from non‐compartmental analyses were compared with previously published reference values [7, 8]. Although EVG trough (C_tau_) values were numerically lower in children than in the adult reference population, the 90% CI of % GLSM ratio included 100, the mean C_tau_ was four‐fold above the 95% inhibitory concentration for wild‐type virus (44.5 ng/ml), and high rates of virologic suppression were observed. Furthermore, as C_max_ and AUC_tau_ were already elevated (but within the adult range), EVG exposures were considered adequate. For COBI and FTC, C_tau_ values were comparable, ensuring adequate efficacy; C_max_ and AUC_tau_ values were consistent with historical data in adults [5]. For TAF, although AUC_tau_ was higher than in adults, C_max_ was comparable because of rapid intracellular clearance; all exposure parameters for TFV were also comparable. In the absence of safety and efficacy concerns, no clinically meaningful differences were observed in EVG, COBI, FTC or TAF exposures in children receiving the low‐dose E/C/F/TAF tablet versus adults receiving E/C/F/TAF in historical studies.

Current treatment options in the USA for children with HIV aged ≥2 years, weighing ≥14–<25 kg, are limited [11, 12]. Younger children may struggle to swallow tablets; therefore, age‐appropriate formulations and smaller tablet sizes may improve acceptability and adherence [10, 12]. The availability of single‐tablet regimens is particularly important for children, in whom a significant treatment burden has been identified as a substantial adherence barrier [11]. The low‐dose E/C/F/TAF tablet measures 16 mm × 7 mm, which is comparable in size to low‐dose bictegravir/F/TAF (B/F/TAF), the smallest single‐tablet regimen currently approved [13]. The tablet was well tolerated by children, as evidenced by a lack of discontinuations or E/C/F/TAF‐related Grade 3 or 4 TEAEs. Importantly, no new safety concerns were identified in younger children compared with Week 48 data reported for Cohorts 1 and 2 [7, 8]. Consistent with previous reports [7, 8, 14], the most common drug‐related TEAEs in this cohort were vomiting and diarrhoea. However, these TEAEs did not lead to discontinuation and were self‐limited.

Low‐dose E/C/F/TAF resulted in high levels of sustained virologic suppression for children with undetectable viral loads at baseline, maintained through 48 weeks of follow‐up. While there was a decline from baseline in absolute CD4 count in participants in Cohort 3, the CD4 absolute count and percentage remained within age‐specific ranges seen in children without HIV and are consistent with the physiological decline in CD4 counts observed with increasing age in populations without HIV in this age group [15]. These results were reinforced by the stable percentage of CD4 cells during the treatment.

Bone and renal safety are particularly important for the paediatric population as ART is administered during a period of intensive growth and maturation [16]. Due to lower plasma TFV exposure, TAF has a better renal and safety profile than tenofovir disoproxil [17]. Overall, there were no signs of negative impact of low‐dose E/C/F/TAF on bone health, with increases in LS and TBLH BMD from baseline during the follow‐up, as expected in growing children regardless of HIV status [18, 19]. Reassuringly, there were no renal TEAEs.

Current guidelines for the use of ART in paediatric patients with HIV recommend bictegravir‐ or dolutegravir‐based combinations as preferred ART for children [11, 20]. Our data show that the E/C/F/TAF low‐dose tablet provides a valuable additional single‐tablet regimen option for this age/weight population. This study also adds to the growing evidence on the positive safety profile of F/TAF‐based regimens, including bone and renal safety, which can be translated to the safety of other preferred single‐tablet paediatric regimens such as B/F/TAF.

## 5 CONCLUSIONS

Overall, our results suggest that low‐dose E/C/F/TAF (90/90/120/6 mg) is a viable single‐tablet regimen with a positive safety and efficacy profile, which expands options for INSTI‐based ARTs in the paediatric population.

## COMPETING INTERESTS

EN, AHG and PK report research funding from Gilead Sciences, Inc., paid to respective institutions. EH and AL declare that they have no competing interests. RS reports research funding paid to institution from Gilead Sciences, Inc., GSK, Merck and Penta; consulting fees from GSK and ViiV Healthcare; and travel support from Enanta and Gilead Sciences, Inc. SCo, RP, VV and KK are employees of, and hold stocks in, Gilead Sciences, Inc. RL was an employee of Gilead Sciences, Inc., at the time of study development. SCr is an employee of Gilead Sciences, Inc., and holds stocks/shares in Alcon, Gilead Sciences, Inc., Novartis and Sandoz. NR reports research funding paid to institution from Gilead Sciences, Inc., GSK, Merck and Penta; and travel support from Gilead Sciences, Inc.

## AUTHORS’ CONTRIBUTIONS

VV and KK designed the study; EN, AHG, PK, EH, RS, AL, VV, KK and NR collected the data; EN, SCo, RL, RP, SCr, VV, KK and NR were responsible for data analysis or interpretation. All authors reviewed and critically revised the manuscript and approved the final draft. All authors are accountable for the accuracy and integrity of the manuscript.

## FUNDING

This study was funded by Gilead Sciences, Inc. (Foster City, California, USA).

## Data Availability

Gilead Sciences, Inc., shares anonymized individual patient data upon request or as required by law or regulation with qualified external researchers based on submitted curriculum vitae and reflecting non‐conflict of interest. The request proposal must also include a statistician. Approval of such requests is at the discretion of Gilead Sciences, Inc., and is dependent on the nature of the request, the merit of the research proposed, the availability of the data and the intended use of the data. Data requests should be sent to DataSharing@gilead.com.
